# Bis(1*H*-imidazol-3-ium) naphthalene-1,5-disulfonate

**DOI:** 10.1107/S1600536812016972

**Published:** 2012-04-25

**Authors:** Bin Wei

**Affiliations:** a Ordered Matter Science Research Center, Southeast University, Nanjing 211189, People’s Republic of China

## Abstract

The asymmetric unit of the title organic salt, 2C_3_H_5_N_2_^+^·C_10_H_6_O_6_S_2_^2−^, consists of an imidazolium cation and half a naphthalene-1,5-disulfonate dianion, completed to the full dianion through an inversion center. N—H⋯S and N—H⋯O hydrogen bonds link cations and anions in the crystal, forming a chain propagating along [101].

## Related literature

For general background to structure phase transitions in ferroelectrics, see: Ye *et al.* (2009 ▶); Zhang *et al.* (2009 ▶).
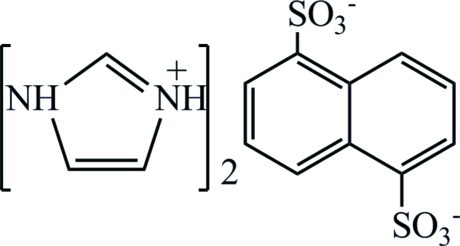


## Experimental

### 

#### Crystal data

2C_3_H_5_N_2_^+^·C_10_H_6_O_6_S_2_^2−^
*M* *_r_* = 424.45
Triclinic, 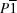

*a* = 6.6764 (13) Å
*b* = 6.7958 (14) Å
*c* = 10.251 (2) Å
α = 93.66 (3)°
β = 103.30 (3)°
γ = 96.77 (3)°
*V* = 447.48 (16) Å^3^
*Z* = 1
Mo *K*α radiation
μ = 0.34 mm^−1^
*T* = 293 K
0.55 × 0.44 × 0.36 mm

#### Data collection

Rigaku SCXmini diffractometer
Absorption correction: multi-scan (*CrystalClear*; Rigaku, 2005 ▶) *T*_min_ = 0.837, *T*_max_ = 0.885
4578 measured reflections
2043 independent reflections
1901 reflections with *I* > 2σ(*I*)
*R*_int_ = 0.025

#### Refinement

*R*[*F* ^2^ > 2σ(*F* ^2^)] = 0.036
*wR*(*F* ^2^) = 0.095
*S* = 1.13
2043 reflections
128 parameters
H-atom parameters constrained
Δρ_max_ = 0.34 e Å^−3^
Δρ_min_ = −0.36 e Å^−3^

### 

Data collection: *CrystalClear* (Rigaku, 2005 ▶); cell refinement: *CrystalClear*; data reduction: *CrystalClear*; program(s) used to solve structure: *SHELXS97* (Sheldrick, 2008 ▶); program(s) used to refine structure: *SHELXL97* (Sheldrick, 2008 ▶); molecular graphics: *SHELXTL* (Sheldrick, 2008 ▶); software used to prepare material for publication: *SHELXTL*.

## Supplementary Material

Crystal structure: contains datablock(s) I, global. DOI: 10.1107/S1600536812016972/bh2419sup1.cif

Structure factors: contains datablock(s) I. DOI: 10.1107/S1600536812016972/bh2419Isup2.hkl

Supplementary material file. DOI: 10.1107/S1600536812016972/bh2419Isup3.cml

Additional supplementary materials:  crystallographic information; 3D view; checkCIF report

## Figures and Tables

**Table 1:** Hydrogen-bond geometry (Å, °)

*D*—H⋯*A*	*D*—H	H⋯*A*	*D*⋯*A*	*D*—H⋯*A*
N2—H2*A*⋯S1^i^	0.86	2.84	3.588 (2)	147
N2—H2*A*⋯O3^i^	0.86	1.90	2.745 (2)	168
N1—H1*A*⋯O2^ii^	0.86	2.09	2.847 (2)	147

Symmetry codes: (i) 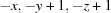; (ii) 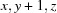.

**Fig. 1.**
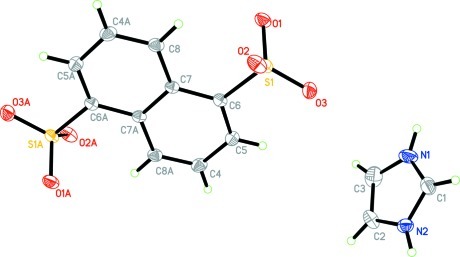
The molecular structure of the title compound, with displacement ellipsoids drawn at the 30% probability level.

**Fig. 2.**
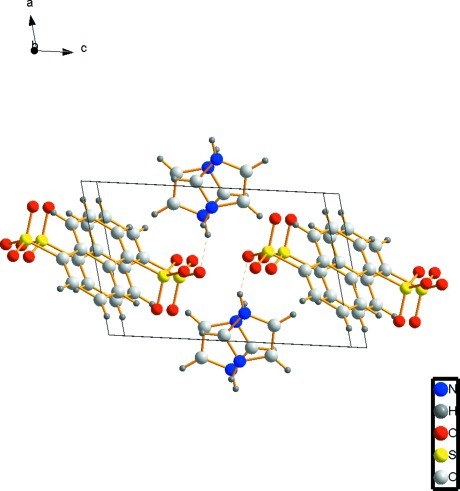
A view of the packing of the title compound, along the b axis. Dashed lines indicate hydrogen bonds.

## supplementary crystallographic information

### Comment

Dielectric-ferroelectric constitute an interesting class of materials, comprising organic ligands, metal-organic coordination compounds, organic-inorganic hybrids and organic salts (Ye *et al.*, 2009; Zhang *et al.*, 2009). Unfortunately, the dielectric constant of the title compound as a function of temperature indicates that the permittivity is basically temperature-independent, below the melting point of the compound. We have found that title compound has no dielectric anomaly from 80 K to 405 K. Herein we describe the crystal structure of this compound.

The asymmetric unit of the title compound consists of an imidazolium cation in general position, and a half naphthalene-1,5-disulfonate anion, close to a inversion center (Fig. 1). The imidazolium ring and the naphthalene ring make a dihedral angle of 69.3°. The cations and anions are connected in the crystal by N—H···S and N—H···O hydrogen bonds, which improve the stability of the crystal structure. These hydrogen bonds link the cations and anions into a chain oriented in the [101] direction (Fig. 2 and Table 1).

### Experimental

The title compound was obtained by the addition of naphthalene-1,5-disulfonic acid (2.88 g, 0.01 mol) to a solution of imidazole (1.36 g, 0.02 mol) in water, in the stoichiometric ratio 1:2. Good quality single crystals were obtained by slow evaporation, after two days (yield: 38%).

### Refinement

All H atoms were placed in geometrically idealized positions and constrained to ride on their parent atoms with C—H = 0.93 Å, N—H = 0.86 Å and with *U*_iso_(H) = 1.2 *U*_eq_(carrier atom).

### Crystal data

**Table:**

2C_3_H_5_N_2_^+^·C_10_H_6_O_6_S_2_^2^^−^	*V* = 447.48 (16) Å^3^
*M**_r_* = 424.45	*Z* = 1
Triclinic, *P*1	*F*(000) = 220
Hall symbol: -P 1	*D*_x_ = 1.575 Mg m^−^^3^
*a* = 6.6764 (13) Å	Mo *K*α radiation, λ = 0.71073 Å
*b* = 6.7958 (14) Å	µ = 0.34 mm^−^^1^
*c* = 10.251 (2) Å	*T* = 293 K
α = 93.66 (3)°	Block, colorless
β = 103.30 (3)°	0.55 × 0.44 × 0.36 mm
γ = 96.77 (3)°	

### Data collection

**Table:**

Rigaku SCXmini diffractometer	2043 independent reflections
Radiation source: fine-focus sealed tube	1901 reflections with *I* > 2σ(*I*)
Graphite monochromator	*R*_int_ = 0.025
CCD_Profile_fitting scans	θ_max_ = 27.5°, θ_min_ = 3.0°
Absorption correction: multi-scan (*CrystalClear*; Rigaku, 2005)	*h* = −8→8
*T*_min_ = 0.837, *T*_max_ = 0.885	*k* = −8→8
4578 measured reflections	*l* = −13→13

### Refinement

**Table:**

Refinement on *F*^2^	Secondary atom site location: difference Fourier map
Least-squares matrix: full	Hydrogen site location: inferred from neighbouring sites
*R*[*F*^2^ > 2σ(*F*^2^)] = 0.036	H-atom parameters constrained
*wR*(*F*^2^) = 0.095	*w* = 1/[σ^2^(*F*_o_^2^) + (0.0312*P*)^2^ + 0.2736*P*] where *P* = (*F*_o_^2^ + 2*F*_c_^2^)/3
*S* = 1.13	(Δ/σ)_max_ = 0.001
2043 reflections	Δρ_max_ = 0.34 e Å^−^^3^
128 parameters	Δρ_min_ = −0.36 e Å^−^^3^
0 restraints	Extinction correction: *SHELXL97* (Sheldrick, 2008), Fc^*^=kFc[1+0.001xFc^2^λ^3^/sin(2θ)]^-1/4^
0 constraints	Extinction coefficient: 0.473 (17)
Primary atom site location: structure-invariant direct methods	

### Fractional atomic coordinates and isotropic or equivalent isotropic displacement parameters (Å^2^)

**Table:**

	*x*	*y*	*z*	*U*_iso_*/*U*_eq_	
N1	0.1864 (3)	0.8251 (3)	0.5703 (2)	0.0502 (5)	
H1A	0.3103	0.8750	0.5689	0.060*	
C1	0.0353 (3)	0.7669 (3)	0.4648 (2)	0.0414 (5)	
H1C	0.0430	0.7727	0.3757	0.050*	
N2	−0.1299 (2)	0.6987 (2)	0.50561 (17)	0.0339 (4)	
H2A	−0.2490	0.6512	0.4542	0.041*	
C2	−0.0821 (4)	0.7151 (3)	0.6423 (2)	0.0429 (5)	
H2C	−0.1709	0.6782	0.6970	0.051*	
C3	0.1172 (4)	0.7945 (4)	0.6829 (3)	0.0561 (7)	
H3B	0.1939	0.8233	0.7715	0.067*	
S1	0.59698 (6)	0.27214 (7)	0.74446 (4)	0.02816 (18)	
O1	0.8155 (2)	0.3366 (2)	0.80260 (13)	0.0360 (3)	
O2	0.5600 (2)	0.0983 (2)	0.64608 (13)	0.0381 (4)	
O3	0.4864 (2)	0.4326 (2)	0.68915 (14)	0.0399 (4)	
C4	0.2276 (3)	0.2277 (3)	1.0055 (2)	0.0349 (4)	
H4A	0.1216	0.2937	1.0241	0.042*	
C5	0.3273 (3)	0.2901 (3)	0.90549 (19)	0.0317 (4)	
H5A	0.2866	0.3968	0.8582	0.038*	
C6	0.4841 (3)	0.1945 (3)	0.87756 (16)	0.0253 (4)	
C7	0.5517 (2)	0.0313 (2)	0.94962 (16)	0.0239 (3)	
C8	0.7149 (3)	−0.0712 (3)	0.92461 (18)	0.0304 (4)	
H8A	0.7835	−0.0309	0.8595	0.036*	

### Atomic displacement parameters (Å^2^)

**Table:**

	*U*^11^	*U*^22^	*U*^33^	*U*^12^	*U*^13^	*U*^23^
N1	0.0278 (8)	0.0370 (10)	0.0858 (15)	−0.0023 (7)	0.0143 (9)	0.0151 (10)
C1	0.0479 (12)	0.0383 (11)	0.0473 (12)	0.0133 (9)	0.0237 (10)	0.0131 (9)
N2	0.0272 (8)	0.0314 (8)	0.0409 (9)	0.0021 (6)	0.0048 (7)	0.0024 (7)
C2	0.0543 (13)	0.0361 (10)	0.0402 (11)	−0.0044 (9)	0.0206 (10)	0.0036 (8)
C3	0.0621 (15)	0.0423 (12)	0.0477 (13)	−0.0113 (11)	−0.0098 (11)	0.0023 (10)
S1	0.0252 (2)	0.0347 (3)	0.0221 (2)	−0.00415 (16)	0.00433 (16)	0.00418 (16)
O1	0.0263 (7)	0.0450 (8)	0.0331 (7)	−0.0064 (5)	0.0048 (5)	0.0061 (6)
O2	0.0400 (8)	0.0454 (8)	0.0260 (7)	−0.0091 (6)	0.0119 (6)	−0.0049 (6)
O3	0.0363 (7)	0.0451 (8)	0.0360 (7)	0.0012 (6)	0.0029 (6)	0.0155 (6)
C4	0.0326 (9)	0.0414 (10)	0.0351 (10)	0.0129 (8)	0.0130 (8)	0.0027 (8)
C5	0.0319 (9)	0.0323 (9)	0.0311 (9)	0.0069 (7)	0.0063 (7)	0.0044 (7)
C6	0.0246 (8)	0.0295 (8)	0.0202 (7)	−0.0008 (6)	0.0047 (6)	0.0000 (6)
C7	0.0210 (7)	0.0287 (8)	0.0200 (7)	0.0000 (6)	0.0038 (6)	−0.0019 (6)
C8	0.0276 (9)	0.0395 (10)	0.0266 (8)	0.0054 (7)	0.0111 (7)	0.0033 (7)

### Geometric parameters (Å, º)

**Table:**

N1—C1	1.301 (3)	O3—S1	1.4601 (15)
N1—C3	1.358 (4)	S1—C6	1.7828 (18)
N1—H1A	0.8600	C4—H4A	0.9300
C1—H1C	0.9300	C4—C8^i^	1.363 (3)
N2—C1	1.312 (3)	C5—H5A	0.9300
N2—C2	1.358 (3)	C5—C4	1.406 (3)
N2—H2A	0.8600	C6—C5	1.369 (3)
C2—C3	1.334 (3)	C6—C7	1.431 (2)
C2—H2C	0.9300	C7—C8	1.422 (2)
C3—H3B	0.9300	C7—C7^i^	1.428 (3)
O1—S1	1.4464 (14)	C8—C4^i^	1.363 (3)
O2—S1	1.4621 (15)	C8—H8A	0.9300
			
C1—N1—C3	109.10 (18)	O1—S1—C6	107.15 (8)
C1—N1—H1A	125.4	O3—S1—C6	106.12 (9)
C3—N1—H1A	125.4	O2—S1—C6	105.93 (8)
N1—C1—N2	108.4 (2)	C8^i^—C4—C5	120.55 (17)
N1—C1—H1C	125.8	C8^i^—C4—H4A	119.7
N2—C1—H1C	125.8	C5—C4—H4A	119.7
C1—N2—C2	108.91 (18)	C6—C5—C4	120.21 (17)
C1—N2—H2A	125.5	C6—C5—H5A	119.9
C2—N2—H2A	125.5	C4—C5—H5A	119.9
C3—C2—N2	106.7 (2)	C5—C6—C7	121.20 (16)
C3—C2—H2C	126.7	C5—C6—S1	118.37 (14)
N2—C2—H2C	126.7	C7—C6—S1	120.39 (13)
C2—C3—N1	106.9 (2)	C8—C7—C7^i^	118.9 (2)
C2—C3—H3B	126.5	C8—C7—C6	123.07 (16)
N1—C3—H3B	126.5	C7^i^—C7—C6	118.00 (19)
O1—S1—O3	112.88 (9)	C4^i^—C8—C7	121.10 (17)
O1—S1—O2	112.73 (9)	C4^i^—C8—H8A	119.5
O3—S1—O2	111.46 (9)	C7—C8—H8A	119.5

Symmetry code: (i) −*x*+1, −*y*, −*z*+2.

### Hydrogen-bond geometry (Å, º)

**Table:**

*D*—H···*A*	*D*—H	H···*A*	*D*···*A*	*D*—H···*A*
N2—H2*A*···S1^ii^	0.86	2.84	3.588 (2)	147
N2—H2*A*···O3^ii^	0.86	1.90	2.745 (2)	168
N1—H1*A*···O2^iii^	0.86	2.09	2.847 (2)	147
N1—H1*A*···O2^iv^	0.86	2.56	3.118 (3)	124

Symmetry codes: (ii) −*x*, −*y*+1, −*z*+1; (iii) *x*, *y*+1, *z*; (iv) −*x*+1, −*y*+1, −*z*+1.
